# Long-Term Morbidity and Health After Early Menopause Due to Oophorectomy in Women at Increased Risk of Ovarian Cancer: Protocol for a Nationwide Cross-Sectional Study With Prospective Follow-Up (HARMOny Study)

**DOI:** 10.2196/24414

**Published:** 2021-01-22

**Authors:** Lara Terra, Maartje J Hooning, Bernadette A M Heemskerk-Gerritsen, Marc van Beurden, Jeanine E Roeters van Lennep, Helena C van Doorn, Joanne A de Hullu, Constantijne Mom, Eleonora B L van Dorst, Marian J E Mourits, Brigitte F M Slangen, Katja N Gaarenstroom, M Carola Zillikens, Tim Leiner, Lizet van der Kolk, Margriet Collee, Marijke Wevers, Margreet G E M Ausems, Klaartje van Engelen, Lieke PV Berger, Christi J van Asperen, Encarna B Gomez-Garcia, Irma van de Beek, Matti A Rookus, Michael Hauptmann, Eveline M Bleiker, Sanne B Schagen, Neil K Aaronson, Angela H E M Maas, Flora E van Leeuwen

**Affiliations:** 1 Department of Psychosocial Research and Epidemiology The Netherlands Cancer Institute Amsterdam Netherlands; 2 Department of Medical Oncology Erasmus University Medical Center Rotterdam Netherlands; 3 Department of Gynaecology Antoni van Leeuwenhoek Amsterdam Netherlands; 4 Department of Internal Medicine Erasmus University Medical Center Rotterdam Netherlands; 5 Department for Gynaecologic Oncology Erasmus University Medical Center Rotterdam Netherlands; 6 Department for Gynaecology Radboud University Medical Center Nijmegen Netherlands; 7 Department of Gynaecology Amsterdam University Medical Centers Amsterdam Netherlands; 8 Department for Gynaecologic Oncology University Medical Center Utrecht Utrecht Netherlands; 9 Department for Gynaecologic Oncology University Medical Center Groningen, University of Groningen Groningen Netherlands; 10 Department for Gynaecology Maastricht University Medical Center Maastricht Netherlands; 11 Department of Gynaecology Leiden University Medical Center Leiden Netherlands; 12 Department Radiology University Medical Center Utrecht Utrecht Netherlands; 13 Family Cancer Clinic Netherlands Cancer Institute Amsterdam Netherlands; 14 Department for Clinical Genetics Erasmus University Medical Center Rotterdam Netherlands; 15 Department for Clinical Genetics Radboud University Medical Center Nijmegen Netherlands; 16 Division of Laboratories, Pharmacy and Biomedical Genetics Department of Genetics University Medical Center Utrecht Utrecht Netherlands; 17 Department for Clinical Genetics Amsterdam University Medical Centers Vrije University Amsterdam Amsterdam Netherlands; 18 Department of Genetics University Medical Center Groningen Groningen Netherlands; 19 Department for Clinical Genetics Leiden University Medical Center Leiden Netherlands; 20 Department of Genetics Maastricht University Medical Center Maastricht Netherlands; 21 Department for Clinical Genetics Amsterdam University Medical Centers University of Amsterdam Amsterdam Netherlands; 22 Brandenburg Medical School Theodor Fontane Institute of Biostatistics and Registry Research Neuruppin Germany; 23 Department of Cardiology Radboud University Medical Center Nijmegen Netherlands

**Keywords:** risk-reducing salpingo-oophorectomy, BRCA1/2, cardiovascular disease, osteoporosis, cognition, health-related quality of life

## Abstract

**Background:**

BRCA1/2 mutation carriers are recommended to undergo risk-reducing salpingo-oophorectomy (RRSO) at 35 to 45 years of age. RRSO substantially decreases ovarian cancer risk, but at the cost of immediate menopause. Knowledge about the potential adverse effects of premenopausal RRSO, such as increased risk of cardiovascular disease, osteoporosis, cognitive dysfunction, and reduced health-related quality of life (HRQoL), is limited.

**Objective:**

The aim of this study is to assess the long-term health effects of premenopausal RRSO on cardiovascular disease, bone health, cognitive functioning, urological complaints, sexual functioning, and HRQoL in women with high familial risk of breast or ovarian cancer.

**Methods:**

We will conduct a multicenter cross-sectional study with prospective follow-up, nested in a nationwide cohort of women at high familial risk of breast or ovarian cancer. A total of 500 women who have undergone RRSO before 45 years of age, with a follow-up period of at least 10 years, will be compared with 250 women (frequency matched on current age) who have not undergone RRSO or who have undergone RRSO at over 55 years of age. Participants will complete an online questionnaire on lifestyle, medical history, cardiovascular risk factors, osteoporosis, cognitive function, urological complaints, and HRQoL. A full cardiovascular assessment and assessment of bone mineral density will be performed. Blood samples will be obtained for marker analysis. Cognitive functioning will be assessed objectively with an online neuropsychological test battery.

**Results:**

This study was approved by the institutional review board in July 2018. In February 2019, we included our first participant. As of November 2020, we had enrolled 364 participants in our study.

**Conclusions:**

Knowledge from this study will contribute to counseling women with a high familial risk of breast/ovarian cancer about the long-term health effects of premenopausal RRSO. The results can also be used to offer health recommendations after RRSO.

**Trial Registration:**

ClinicalTrials.gov NCT03835793; https://clinicaltrials.gov/ct2/show/NCT03835793.

**International Registered Report Identifier (IRRID):**

DERR1-10.2196/24414

## Introduction

### Background

Female *BRCA1* germline mutation carriers have a cumulative risk of 44% to develop ovarian cancer before the age of 80 years. For *BRCA2* mutation carriers, this cumulative risk is 17% [1]. In the absence of an effective screening method for ovarian cancer, risk-reducing salpingo-oophorectomy (RRSO) is advised at an age when there is a high risk of developing ovarian cancer (before the age of 35-40 years for *BRCA1* mutation carriers and at the age of 40-45 years for *BRCA2* mutation carriers) provided that the woman no longer has a wish to have children [2,3]. During this procedure, both ovaries and fallopian tubes are removed. Since 2009, the uptake of RRSO in *BRCA1/2* mutation carriers in the Netherlands has increased greatly from 81% to 98% currently [4-6]. While RRSO at the recommended age decreases the risk of ovarian cancer by around 96% [7,8], it also initiates premature surgical menopause owing to a sudden drop in the levels of ovarian hormones. Whether RRSO also reduces the risk of breast cancer is currently debated. Various early studies have reported a risk reduction of 50%, but this is a substantial overestimation owing to bias [8-11].

The relationship between early menopause (either surgically induced or due to premature ovarian insufficiency [POI]) and noncancer health outcomes, including cardiovascular disease (CVD), osteoporosis, cognition, and health-related quality of life (HRQoL), has been examined previously. However, most of the published studies had a limited follow-up (median <5 years), which is too short to establish an association with long-term health effects [12-15]. Additionally, hormone replacement therapy (HRT) use, cancer treatment, and lifestyle may alter the potential association of RRSO with noncancer health outcomes. *BRCA1/2* mutation carriers may have been treated for breast cancer, which can be cardiotoxic and potentially have a negative impact on the brain [16-19]. Lastly, women with a high familial risk of breast/ovarian cancer may have a healthier lifestyle than age-matched women without a family history of breast cancer, rendering translation of results of previous research on early menopause to this specific population difficult [20].

### Early Menopause and Cardiovascular Risk

Before the age of 50 years, female sex hormones, such as estrogen and progesterone, are likely to protect women against CVD as they have atheroprotective properties [21,22]. Previous studies have shown that women with POI are at higher risk of developing CVD and that earlier age at menopause is associated with a higher cardiovascular mortality rate [23-26]. Additionally, a higher risk of CVD has been observed after *surgical* menopause compared with *natural* menopause [27]. However, age at surgical menopause was often not specified or surgery was performed at later ages than the recommended young ages for RRSO in *BRCA1/2* mutation carriers. Thus, information about the cardiovascular risk of *early* surgical menopause is still limited [27-29]. Furthermore, it is possible that early loss of ovarian hormones is not the *cause* of the higher cardiovascular disease risk in women with POI. The reverse causality hypothesis postulates that POI is the result of accelerated vascular aging, which would also explain a statistical (noncausal) association between earlier natural menopause and increased CVD risk [30]. If this is true, no elevated CVD risk would be expected in women with early surgical menopause due to RRSO.

### Early Menopause and Bone Health

Reproductive hormones play a role in maintaining bone health. Estrogen suppresses bone resorption and stimulates bone formation [31]. Postmenopausal women aged 50 to 55 years have a higher incidence of hip fractures than age-matched premenopausal women, and among postmenopausal women, the incidence of hip fractures rises with age [32]. However, data on the long-term effect of early (surgical or natural) menopause on bone health are limited owing to short follow-up periods.

### Early Menopause and Cognition

The neuroprotective effects of estrogens, progestogens, and androgens have been described in several studies [33]. Rocca et al observed that women who underwent premenopausal oophorectomy (before 56 years of age) for benign reasons had a higher risk of cognitive impairment than age-matched women without oophorectomy [34]. There was a trend for more severe cognitive impairment with younger age at oophorectomy for both unilateral and bilateral oophorectomies. However, information on *BRCA1/2* mutation carriership was not provided [34]. Additionally, Bove et al observed a faster decline in global cognition in women with an earlier age at surgical menopause [14]. Thus, while there are some data on the effect of surgical menopause on cognition, research on *BRCA1/2* mutation carriers is limited.

### Early Menopause and HRQoL

RRSO may have an important impact on HRQoL, including generic issues, such as physical, role, and emotional functioning, as well as cancer-related anxiety, sexual functioning, and menopause-specific symptoms [12,35,36]. However, it is not known whether these effects persist over time. A recent study with a mean follow-up of 7.9 years after RRSO found that moderate to severe menopausal complaints were still present in almost 70% of women who underwent premenopausal RRSO, without any improvement over time [37]. The magnitude of the effect of RRSO on sexual functioning and endocrine symptoms is modulated by HRT use [38,39]. However, HRT use is contraindicated for *BRCA1/2* mutation carriers with a history of breast cancer.

The number of postmenopausal years is associated with an increased prevalence of vulvovaginal atrophy [40]. Atrophy of the urethral epithelium may lead to urinary incontinence. To our knowledge, no research has been conducted on the association between early surgical menopause and urinary incontinence, although this generally has a severe impact on sexual functioniƒng and quality of life [41,42].

**Figure 1:**
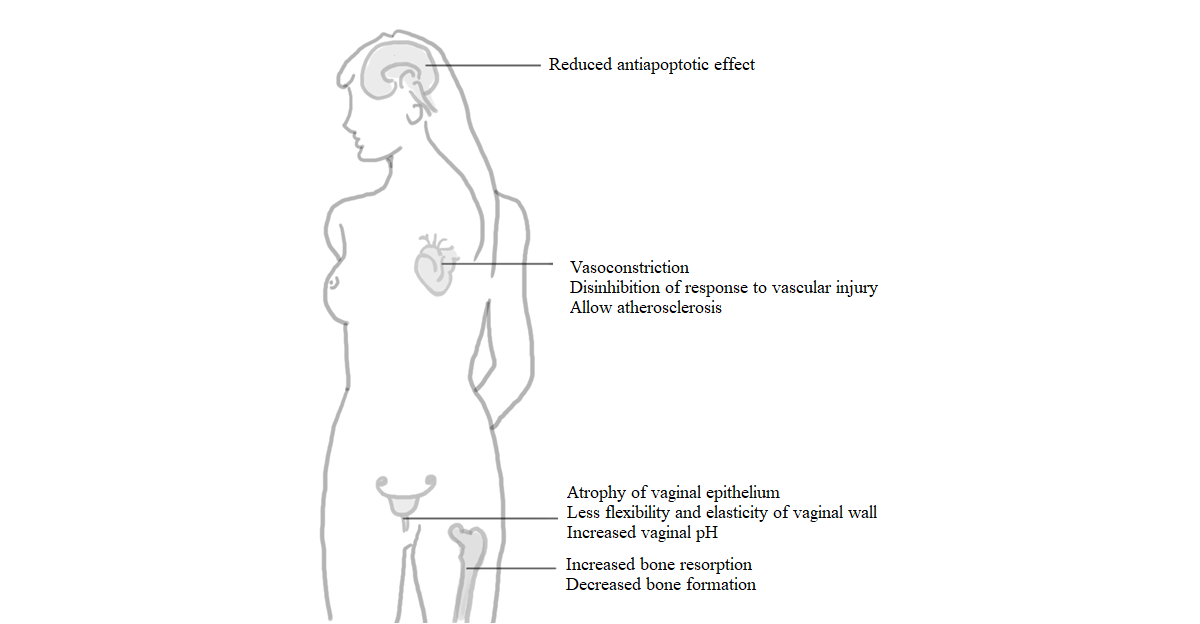
Overview of the effects of reduced estrogen levels on various organ systems.

Figure 1 provides a schematic overview of the effects of a decrease in estrogen levels due to menopause on various organ systems.

A recent Cochrane systematic review on the benefits and harms of RRSO in women with a *BRCA1/2* mutation stated that no conclusions can be drawn regarding bone fracture incidence and HRQoL, and that further research is needed. CVD and cognition were not included as outcomes in this review, probably because of the paucity of studies [43]. Therefore, the aim of the HARMOny study (Health After eaRly Menopause due to Oophorectomy) is to investigate the long-term health effects of RRSO. We will conduct a cross-sectional study with prospective follow-up nested in a large Dutch cohort. We hypothesize that women with surgically induced premature menopause are at higher risk of subclinical CVD, compromised bone health and osteoporosis, reduced HRQoL, and impaired cognitive functioning, compared to age-matched women with natural menopause after the age of 50 years. Depending on the results, this may lead to active surveillance of the above health problems to improve long-term health and HRQoL in women who have undergone early RRSO.

## Methods

### Study Aim

The primary aim of our study is to assess the long-term health effects of RRSO on subclinical CVD, bone mineral density (BMD), and cognitive functioning in women at increased familial risk of breast or ovarian cancer mainly due to a *BRCA1/2* mutation. Our secondary study aims are to assess overall HRQoL, endocrine symptoms, such as hot flashes and night sweats, sexual functioning, urogenital problems, and the prevalence of cardiovascular risk factors.

### Recruitment

We will include 500 women who have undergone RRSO before the age of 45 years, with a follow-up period of at least 10 years (the early RRSO group). We will compare them with 250 women from the same cohort (frequency matched on current age) who have not undergone preventive RRSO or have undergone RRSO at the age of 55 years or above (the late/non-RRSO group). The exclusion criteria are a metastatic disease; a serious physical comorbidity or psychiatric disorder; a language barrier; a metal cardiac valve; a bare metal coronary stent, since the reflection of the stent would interfere with the computed tomography scan; and natural POI before the age of 40 years. A history of nonovarian cancer, including breast cancer, is not a reason to be excluded.

Eligible women who are alive will be invited for a clinic visit and questionnaire survey that will assess CVD status, BMD, cognitive functioning, and HRQoL. For eligible women who have died, we will collect information on the causes of death to examine selection bias.

Participants will be selected from the dynamic nationwide HEBON study cohort, a national collaboration on HEreditary Breast and Ovarian cancer in the Netherlands, with women tested for *BRCA1/2* germline mutations included since 1997 by all eight Dutch University Medical Centers (UMCs) and the Netherlands Cancer Institute (NKI). The cohort currently consists of over 3600 proven female *BRCA1/*2 germline mutation carriers. Participants have given informed consent for linkage with the Netherlands Cancer Registry and the Dutch Nationwide Pathology Database (PALGA), which can provide information on all prophylactic mastectomies and salpingo-oophorectomies performed in the Netherlands since 1991. This provides us with nearly complete information on the occurrence of RRSO. Data on lifestyle, family history, reproductive factors, and hormone use have been collected from HEBON participants through questionnaires [44].

**Figure 2:**
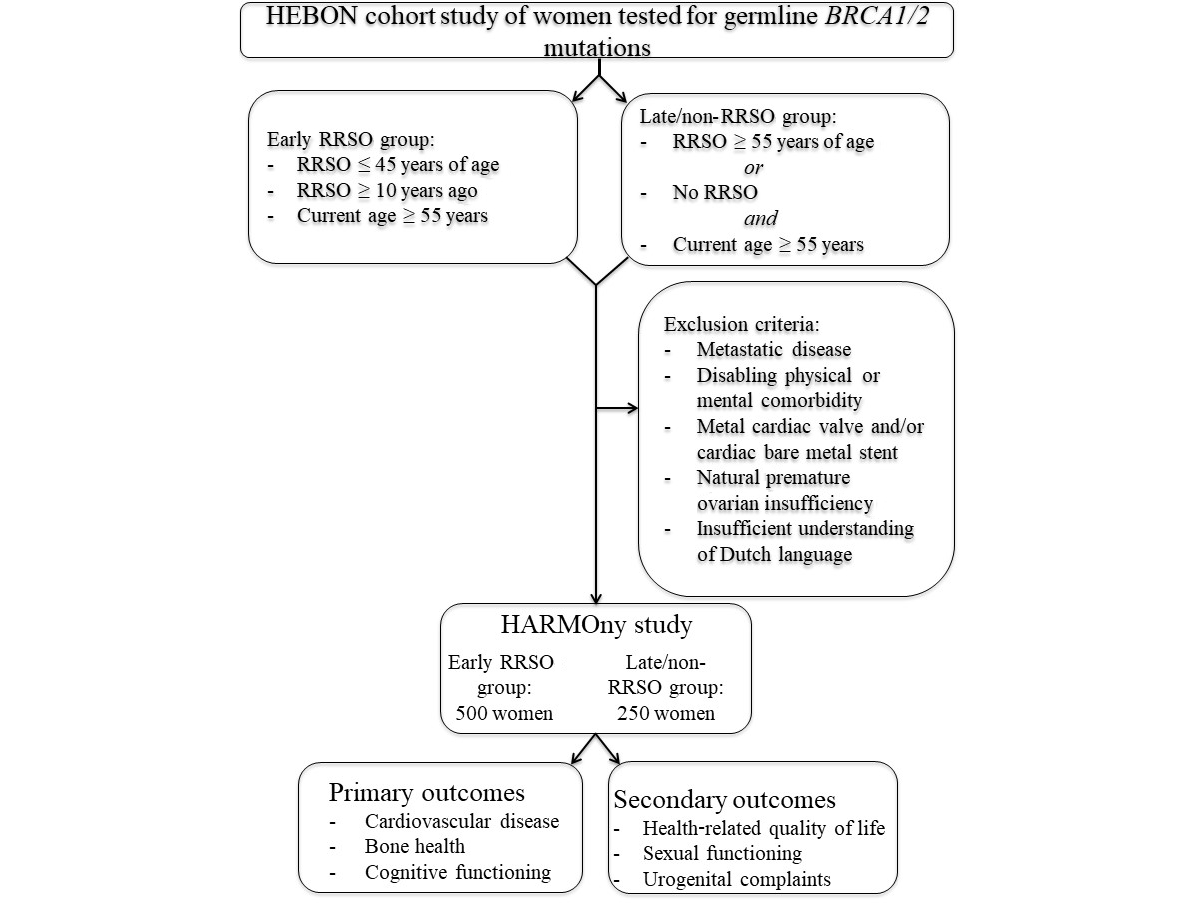
Schematic overview of the eligibility criteria.

Figure 2 provides a schematic overview of the patient selection and eligibility criteria of our study population.

Participants will be asked to complete an online questionnaire and an online cognitive assessment, and they will be asked to visit the outpatient clinic for a cardiovascular and bone health assessment. We will assess several surrogate endpoints of cardiovascular health, that is, coronary artery calcium (CAC) score (CAC scoring), pulse wave velocity (PWV), advanced glycation end products (AGEs), anthropometric measurements, and blood sampling. We will determine nonfasting blood levels of lipids, glucose, HbA_1c_, high-sensitivity cardiac troponin, and high‐sensitivity C‐reactive protein. BMD is assessed by a dual-energy X-ray absorptiometry (DXA) scan and laboratory blood sampling, and cognitive functioning is assessed by the Amsterdam cognition scan (ACS).

The online questionnaire is divided into the following several topics: general cardiovascular history, family history of CVD, traditional CVD risk factors (ie, diabetes, hypertension, and cholesterol), lifestyle (ie, smoking, alcohol use, and physical activity), female-specific risk factors (hypertensive pregnancy complications, gestational diabetes, contraceptives use, and HRT use, ie, type, duration, and timing), history of fractures, calcium intake, vitamin D supplement use, glucocorticoid use, family history of osteoporosis/fractures, history of inflammatory diseases (rheumatoid disease and thyroid disorders), and perceived cognitive problems. We will assess generic HRQoL with the SF-36 health survey [45,46] and the body image items of the European Organization for Research and Treatment of Cancer (EORTC) QLQ-BR23 [47,48]. Cancer worries will be measured with the eight items adapted from Lerman et al [49-51]. To assess menopausal symptoms, we will employ the 18-item Functional Assessment of Cancer Therapy-Endocrine Subscale (FACT-ES) and the Hot Flush Rating Scale (HFRS) [52,53]. To assess urogenital problems, the Urogenital Distress Inventory (UDI-6) and Incontinence Impact Questionnaire (IIQ-7) [54] will be used. The Sexual Activity Questionnaire (SAQ) [55] will be used to assess sexual functioning. For patients with a breast cancer history, either before or after RRSO, we will collect information on radiotherapy (yes/no and radiation fields), chemotherapy and immunotherapy regimens, and endocrine treatment from medical records.

Measurements of the CAC score as calculated by coronary calcium scoring will be performed according to standardized local protocols at the various UMCs using the Agatston score (AS) and the Multi-Ethnic Study of Atherosclerosis (MESA) coronary heart disease risk score calculator [56,57]. A disadvantage of CAC scoring might be the use of ionizing radiation. However, the radiation dose in the CAC protocol is as low as 1 mSv, which is not expected to be harmful [58]. All patients in our study are aged 55 years or above; thus, the radiation-induced risk of breast cancer from CAC scoring will be negligible with the current protocol [59,60].

The PWV will be assessed with an ambulatory arteriograph (TensioMed Kft) by the research physician. The arteriograph uses an oscillometric occlusive method, with an upper arm cuff to measure the time interval between the peak of the first systolic wave and the peak of the reflected systolic wave (return time). The device has been validated in numerous studies [61-65].

With aging, the end products of nonenzymatic modification of proteins, lipids, and nucleic acids (AGEs) accumulate endogenously in the serum and tissues. Levels of AGEs in the skin are measured noninvasively by standardized autofluorescence and can predict future cardiovascular events [66,67]. Skin autofluorescence is a quick and noninvasive surrogate marker of tissue accumulation of AGEs [68-70]. AGEs will be measured using the AGE Reader (DiagnOptics Technologies BV; software V 2.3.0.7) on the forearm.

Bone health will be assessed by a DXA scan, a vertebral fracture assessment, and blood levels of bone turnover markers (BTMs) for osteoclast and osteoblast activity (beta-carboxy-terminal collagen crosslinks [β-CTX] and N-terminal procollagen type 1 [P1NP]). For logistic reasons, it is not possible to get fasting blood samples for every woman. Since β-CTX values are only valid in fasting blood samples, we will assess this BTM only in those women for whom we have fasting blood samples. P1NP will be measured in all samples.

Lastly, women will receive an invitation to fill out the online ACS, which has recently been developed and validated [71]. The ACS is an easy to use tool to obtain online measures of various cognitive abilities [72]. The online test battery is based on seven traditional neuropsychological tests and covers the following domains: verbal memory, attention, executive functioning, information processing speed, and motor functioning.

Table 1 provides an overview of the tests that will be performed [58,61-63,66,67,69,71-77]. Table 2 provides an overview of the questionnaires used [45-50,52-55].

We will examine the effect of age at RRSO on the risk of breast cancer, contralateral breast cancer, and ovarian cancer, as well as associations between RRSO and the prognosis of subsequent breast cancer and ovarian cancer. Additionally, after completion of the cross-sectional study, we will perform longitudinal follow-up every 4 to 5 years to evaluate incidence rates of outcomes of major interest, such as ischemic heart disease, and evaluate changes in outcomes (CAC, PWV, BMD, and cognitive functioning as assessed by the ACS) over time between the early RRSO group and the late/non-RRSO group. Moreover, with the anticipated prospective follow-up of our study population, we will look at mortality of cardiovascular disease.

All data, including blood and DNA samples, will be stored for 30 years to enable prospective longitudinal follow-up of the study population on the long-term health effects of RRSO. The stored blood samples will allow for future international studies, for example, those on the modifying effects of genetic factors, such as single nucleotide polymorphisms, on the outcomes of interest, and the novel cardiovascular markers or BTMs for these outcomes. All blood samples will be frozen and stored at −80°C at the NKI.

**Table 1:** Overview of the tests that will be performed.

Aim of the test	Data collection method and references	Outcome variables	Justification
Cardiovascular health	Coronary artery calcium (CAC) scoring [58,73]	Score according to Agatston	Measurements of the CAC score as calculated by coronary calcium scoring will be performed according to standardized local protocols at the various University Medical Centers using the Agatston score (AS) and the MESA^a^ coronary heart disease risk score calculator.
Cardiovascular health	Pulse wave velocity (PWV) [61-63]	In meters/second	The PWV will be assessed with an ambulatory arteriograph (TensioMed Kft) by the research physician. The arteriograph uses an oscillometric occlusive method, with an upper arm cuff to measure the time interval between the peak of the first systolic wave and the peak of the reflected systolic wave (return time).
Cardiovascular health	Advanced glycation end products (AGEs) [66,67,69]	In arbitrary units (AU)	With aging, the end products of nonenzymatic modification of proteins, lipids, and nucleic acids (AGEs) accumulate endogenously in the serum and tissues. Levels of AGEs in the skin are measured noninvasively by standardized autofluorescence and can predict future cardiovascular events. AGEs will be measured using the AGE Reader (DiagnOptics Technologies BV; software V 2.3.0.7).
Cardiovascular health	Anthropometric measurements	Blood pressure in mmHg Pulse frequency per minute Waist (in cm)/hip (in cm) ratio BMI in kg/m^2^	Predictors of future cardiovascular events.
Cardiovascular health	Laboratory blood sampling	Nonfasting blood levels for lipids (total cholesterol, high-density lipoprotein cholesterol, low-density lipoprotein cholesterol, and triglycerides), glucose, HbA_1c_, high-sensitivity cardiac troponin, high‐sensitivity C‐reactive protein	Predictors of future cardiovascular events.
Bone health	Dual-energy X-ray absorptiometry (DXA) scan of the lumbar spine and hip	BMD^b^ values in g/cm^2^ T and Z scores Presence of osteopenia (defined as T-scores of −1 to −2.5) Presence of osteoporosis (defined as T-scores of ≥−2.5)	The DXA scan is most widely used in clinical practice to screen for osteoporosis and is regarded as the “gold standard.”
Bone health	Vertebral fracture assessment (VFA)	Vertebral height reduction in percentage Presence of clinical and nonclinical vertebral fractures	There is a strong additive value of VFA compared with DXA alone [74-76].
Bone health	Laboratory blood sampling [77]	Bone formation by P1NP^c^, mean value in ng/mL Bone resorption by β-CTX^d^, mean value in pg/mL Level of 25-hydroxyvitamin D in the serum in nmol/L	Bone turnover markers (BTMs) for osteoclast and osteoblast activity (β-CTX and P1NP). For logistic reasons, it is not possible to get fasting blood samples for every woman. Since β-CTX values are only valid in fasting blood samples, we will assess this BTM only in those women for whom we have fasting blood samples. P1NP will be measured in all samples.
Cognitive functioning	Amsterdam cognition scan (ACS) [71]	Verbal memory Attention Executive functioning Information processing speed Motor functioning	The ACS is an easy-to-use tool to obtain online measures of various cognitive abilities [72]. The online test battery is based on seven traditional neuropsychological tests.

^a^MESA: Multi-Ethnic Study of Atherosclerosis.

^b^BMD: bone mineral density.

^c^P1NP: N-terminal procollagen type 1.

^d^β-CTX: beta-carboxy-terminal collagen crosslinks.

**Table 2:** Overview of the questionnaires.

Aim of the questionnaire	Topics
Cardiovascular health	General cardiovascular history Cardiovascular disease risk factors (ie, obesity, diabetes, hypertension, and dyslipidemia) Lifestyle (ie, smoking, alcohol use, and physical activity) Female-specific risk factors (ie, hypertensive pregnancy complications, gestational diabetes, contraceptive use, and hormone replacement therapy use) Nontraditional risk factors (history of inflammatory diseases, ie, rheumatoid disease)
Bone health	History of fractures Calcium intake Vitamin D supplements Glucocorticoid use Family history of osteoporosis/fractures History of inflammatory diseases (ie, rheumatoid disease and thyroid disorders)
Cognitive health	Perceived cognitive problems
Health-related quality of life [45-50,52-55]	SF-36 health survey Body image items of the European Organization for Research and Treatment of Cancer (EORTC) QLQ-BR23 Cancer worries; eight items adapted from Lerman et al The 18-item Functional Assessment of Cancer Therapy-Endocrine Subscale (FACT-ES) measure of endocrine symptoms Hot Flush Rating Scale (HFRS) Urogenital Distress Inventory (UDI-6) Incontinence Impact Questionnaire (IIQ-7) Sexual Activity Questionnaire (SAQ)

### Statistical Analysis

We will analyze differences in the cardiovascular risk profile, BMD, and neurocognitive functioning between the early RRSO group and the late/non-RRSO group using multivariable linear regression analyses and logistic regression analyses with clinically relevant cutoff points for the outcomes of interest. The analyses will be adjusted for confounders, including lifestyle, reproductive factors, and medication use, where applicable. For the analysis of patient-reported outcomes, such as HRQoL, sexual functioning, and menopausal symptoms, we will use mixed effect models.

We expect approximately 40% of the women to have a breast cancer history and will perform the analyses separately in women with and those without a breast cancer history, as breast cancer treatment may affect outcomes. Additional subgroup analyses will be performed according to HRT use (yes/no), *BRCA1/2* mutation status, age at RRSO (<40 years/≥40 years), and time since RRSO (10-15 years/>15 years).

### Sample Size Calculation

We based our sample size calculations for cardiovascular status on subclinical atherosclerosis by assessing the differences in the CAC score between women in the early RRSO group and women in the late/non-RRSO group. We chose a clinically relevant cutoff for the CAC score of 100 Agatston units (AU). According to the MESA trial, 10% of subjects between 55 and 65 years of age exceed this value [78]. For sample size estimation, we hypothesized that 20% of the early RRSO group exceeds this value of 100 AU. In logistic regression with an elevated CAC score (>100) as the outcome and with 250 women in the late/non-RRSO group and 500 women in the early RRSO group, we have 80% power to detect an odds ratio of 1.9 at an α value of 5%.

With 750 women in our study, we will be able to detect a difference of 0.07 g/cm^2^ in BMD between the early RRSO and late/non-RRSO groups, as observed between women with premature and normal menopause and assuming an SD of 0.15 [79]. Statistical power for analysis of cognitive functioning is based on a previous neuropsychological study on the effects of oophorectomy on cognitive impairment and dementia [34]. This study reports relevant differences in 427 women with bilateral oophorectomy before the age of 49 years, compared to age-matched women without oophorectomy, with a median follow‐up of 25 years. As our neurocognitive tests are more sensitive than the tests screening for dementia, we expect to be able to detect smaller differences in cognitive functioning and distinguish between different cognitive domains, and with 750 participants, we have 90% power to detect an effect size of 0.25 with a *P* value set at .05 (two tailed). To detect a relevant difference between groups for the various HRQoL measures, we will use a half SD (an effect size of 0.5) to define clinically relevant group differences (over time). This requires about 67 patients per group. The available sample will be more than adequate to conduct subgroup analyses as well.

## Results

This study was funded by the Dutch Cancer Society in 2016. The Institutional Review Board of the Netherlands Cancer Institute approved this nationwide multicenter study in July 2018, and local approval from the participating sites followed in the months thereafter.

This study will be conducted according to the standards of Good Clinical Practice, in agreement with the principles of the Declaration of Helsinki and with the Dutch law as stated in the Medical Research Involving Human Subjects Act (WMO). The study has been approved in writing by the Medical Ethics Committee of the Antoni van Leeuwenhoek/Netherlands Cancer Institute (AVL/NKI) to be conducted in all nine University Medical Centers and the Antoni van Leeuwenhoek and has been registered at “CCMO Toetsingonline” from the Dutch Central Committee on Research involving Human Subjects (file number NL63554.031.17) and on ClinicalTrials.gov (NCT03835793). Written informed consent will be collected from the participants.

In February 2019, we included our first participant, and as of November 2020, we had enrolled 364 participants in our study. Of these 364 participants, 228 are in the early RRSO group and 136 are in the late/non-RRSO group. Recruitment of participants is currently ongoing. Results will be disseminated through peer-reviewed publications and will be incorporated in follow-up guidelines.

## Discussion

To our knowledge, this study is the first to assess the long-term noncancer health outcomes of early surgical menopause in women with a high familial risk of ovarian cancer, focusing on CVD, bone health, cognition, and HRQoL. The issue is important as RRSO at the age of 35-45 years is an increasingly common procedure in *BRCA1/2* mutation carriers, with an estimated uptake in the Netherlands of 81% to 99% [4,5]. We have designed a nationwide, multicenter, multidisciplinary, cross-sectional study with prospective follow-up, comparing 500 women who have undergone RRSO before 45 years of age (more than 10 years of follow-up) with 250 women who have undergone RRSO after 55 years of age or who have not undergone RRSO. The unique features of our study are near complete information on the occurrence of RRSO in the cohort and long-term follow-up of all cohort members, allowing the selection of the eligible population for this study at least 10 years after RRSO. Our study is nested in a cohort of women with a high familial risk of breast and ovarian cancer, which will allow us to evaluate potential differences in disease risks between our study population and the entire cohort in order to quantify any survivorship and selection bias and to adequately interpret the magnitude of our effect sizes.

At present, it is still unclear whether early surgical menopause can be considered as a clinically relevant risk factor for CVD. During normal menopausal transition, CVD risk increases [23,26,78,80]. Women with an early natural menopause (<40 years) are at higher risk of developing CVD, and they have a higher CVD mortality than age-matched peers [23-26]. However, it has been debated whether this association is causal, and it has been speculated that this might also be explained by early vascular aging predisposing to both POI and cardiovascular disease [30,81]. If this is true, an artificial early menopause might not be associated with increased CVD risk. In support of this, a recent study by Krul et al showed that cancer treatment–induced POI did not affect CVD risk [82]. This renders it even more interesting to investigate CVD risk in women with surgically induced early menopause. To date, this has been addressed in only a few studies, with inconsistent results and with a median follow-up of at the most 5 years, which is too short considering potential delayed effects of estrogen deprivation and the long life expectancy of these women. Furthermore, previous studies vary with respect to HRT use and age at surgery [27-29].

The association between early surgical menopause and long-term bone health has also been rarely examined. Studies were inconclusive regarding decreased BMD and increased fracture risk, and whether these risks remain increased over time [83,84]. In addition, the effect of early RRSO on cognition has not yet been examined. However, both unilateral and bilateral oophorectomies at later ages have been associated with cognitive impairment and dementia, suggesting neuroprotective effects of estrogen on the brain. These effects appear to be age dependent [34,85].

After menopause, women more often report sexual and urogenital problems, such as urinary incontinence, recurrent urinary tract infections, impaired libido, and dyspareunia, which interfere with HRQoL. Women with POI experience these problems even more [86,87]. We do not know if these results can be generalized to surgical early menopause. Studies on long-term HRQoL after RRSO are limited and highly biased by HRT use.

One of the major strengths of our study is that it will be the first to examine the health effects of premenopausal RRSO at less than 45 years with a follow-up of over 10 years compared to controls with natural menopause. Additionally, owing to the large number of participants, we will have sufficient power for subgroup analyses. A limitation of our study could be survivor bias. Owing to the inclusion criterion of at least 10 years of follow-up, patients may already have died of relevant study outcomes before study inclusion. However, as we conduct the cross-sectional study within a well-characterized cohort, we can assess the extent of such bias by collecting cause of death information for potentially eligible but already deceased women.

This initial cross-sectional study will provide prevalence rates of noncancer outcomes. Therefore, we will follow the study cohort prospectively to obtain incidence rates of outcomes of interest and evaluate changes in outcomes over time.

To summarize, the current literature lacks information on long-term noncancer outcomes in women at increased risk of breast/ovarian cancer who have undergone early oophorectomy. With this study, we will be able to provide insights into the prevalence and severity of CVD, osteoporosis, cognitive impairment, and HRQoL effects after RRSO. This knowledge will yield evidence-based information for women and for health care providers about the long-term effects of early RRSO. The results can be incorporated in decision-support tools about risk-reducing surgeries in *BRCA1/2* mutation carriers considering RRSO and can be used to offer individualized counseling about additional screening and interventions for noncancer diseases or complaints after RRSO.

## Appendix

Multimedia Appendix 1 Peer-review report by KWF.

## Abbreviations

ACS: Amsterdam cognition scan
AGEs: advanced glycation end products
AS: Agatston score
AU: Agatston units
BMD: bone mineral density
BRCA: breast cancer gene
BTM: bone turnover marker
CAC: coronary artery calcification
CVD: cardiovascular disease
DXA: dual-energy X-ray absorptiometry
HEBON: HEreditary Breast and Ovarian cancer in the Netherlands
HRQoL: health-related quality of life
HRT: hormone replacement therapy
NKI: Netherlands Cancer Institute
POI: premature ovarian insufficiency
PWV: pulse wave velocity
RRSO: risk-reducing salpingo-oophorectomy
UMC: University Medical Center
