# Supplementary material for: Long-Term Morbidity and Health After Early Menopause Due to Oophorectomy in Women at Increased Risk of Ovarian Cancer: Protocol for a Nationwide Cross-Sectional Study With Prospective Follow-Up (HARMOny Study)
Source: JMIR Res Protoc. 2021 Jan 22;10(1):e24414. doi: 10.2196/24414 (PMC7864779; doi:10.2196/24414)
Supplement: Multimedia Appendix 1 [file resprot_v10i1e24414_fig0.pdf]

Bijlage: Referentencommentaar

Project nummer: 2016-1 / 10164
Project leider: Prof. Flora van Leeuwen

External Review(s) – Exploration

**Reviewer 1**		
	**Overall Score**	**Justification**
**Relevance**	High Relevance	The quality of the cohort data as well as the ability to link to national data for outcomes makes me highly enthusiastic. The investigators have assembled a team with relevant clinical and epidemiological expertise to conduct the clinical examination for the second set of aims. The PI has a long track record of conducting highly important research. Currently, there is limited evidence to help guide high risk women as to the optimal age of risk reducing oophorectomy.
**Scientific Quality**	High Scientific Quality	The record linkage and national registry ensure the high quality of aim 1. Aim 2 will use a subcohort of women for clinical measurements. The measurements selected are sound and the team in place has the relevant expertise.
**Feasibility Workplan**	High Feasibility	The time line and work scope to collect new data mainly for Aim 2 seems reasonable. There are a number of statistical analyses that will require frequent input on outcome definitions and resolutions across the team. The plan for regular communication is essential to carry out to ensure that work is completed within the grant period. Analyses fully characterizing the women that participate in Aim 2 will be essential to understand any response bias related to participation in Aim 2.
**Critical Issues**	No	There are no major critical issues. The team of investigators have a long track record conducting sound, unbiased analyses and the quality of the national data is high.
**Suggestions for Improvement**		Given the space constraints, the issues involved with missing outcome data and integration of results from both aims into clinical decision tools was not covered in detail.<br>Would be important to consider how the sample selection in Aim 2 will be appropriately weighted with the results from Aim 1 for clinical risk assessment.

**Reviewer 2**		
	**Overall Score**	**Justification**

**Relevance**	High Relevance	A huge increase in high-risk women being identified has resulted from increasing awareness/novel BRCA testing strategies of OC/triple-negative BC patients and high prevalence populations/falling costs. Three-fourths of these women undergo RRSO. While fairly robust estimates of OC risk reduction are available and it would be possible to obtain BC risk/ survival estimates from analysis of the 10,000 IBCCS cohort, the project will provide unique objective data on CVD/bone health/congnitiion following premenoausal RRSO which are lacking but critical to decision making.
**Scientific Quality**	High Scientific Quality	The project addresses a highly relevant problem. Key strengths include (1) use of established cohorts (HEBON/IPO) (2) availability of baseline data to assess eligibility/estimate number of recruits (3) adequate sample size (4) completeness of BC/CVD/survival data through linkage to multiple national databases with additional follow-up via general practitioners/patients (4) validated objective endpoints for CVD/bone health/cognitive ability/HRQoL (5) novel features - comprehensive/validated online cognitive function test, measurement of CVD markers such as CIMT, PWV (6) exploration of BAC on mammograms
**Feasibility Workplan**	Medium Feasibility	The arrangements/milestones for protocol development, ethics approval, data linkages, centre set up are adequate.<br>Concerns (1) assumption in WP3 that all consented patients will complete HRQoL, online cognitive test and CVD measurements. It is likely that some will miss one or the other but current milestones do not address these possibilities. (2) The number of patients who will need to travel to another centre for risk marker CVD assessment needs to be estimated and addressed.
**Critical Issues**	Yes	This is not a major issue but WP2 and WP3 are heavily dependent on the ability / commitment of a Phd student as opposed to a postdoctoral epidemiologist/researcher. The PIs of WP2 and 3 have planned a weekly supervisory meeting of the appointed physician researcher in the 1st year and two-weekly meetings thereafter. These supervisions and early identification of any issues with set up and data collection are critical to the success of the project.
**Suggestions for Improvement**		There is not adequate clarification of the study on BAC assessment on mammograms. Would suggest that the most recent mammogram is ultilised in these assessments so that it is contemporaneous to other measurements. It would be also worthwhile to score serial annual mammograms before and after RRSO to estimate onset, progression and correlate any acceleration with time from premenopausal RRSO in cases and natural menopause/post age 55 RRSO in controls.
